# Correlation Between Hypertension, C-Reactive Protein and Serum Uric Acid With Psychological Well-being

**DOI:** 10.5812/ircmj.17205

**Published:** 2014-07-05

**Authors:** Ali Maleki, Saeid Samandari, Osvaldo Almeida, Scott Reza Jafarian Kerman, Mahdi Abdolvand, Farshid Aliyari, Saeid Foroughi

**Affiliations:** 1 School of Medicine, Lorestan University of Medical Sciences, Khorramabad, IR Iran; 2 Psychiatry Research Center, Isfahan University of Medical Sciences, Isfahan, IR Iran; 3 School of Psychiatry and Clinical Neurosciences, University of Western Australia, Crawley, Australia; 4 Students Scientific Research Center, Tehran University of Medical Sciences, Tehran, IR Iran; 5 Azna Health Services Center, Lorestan University of Medical Sciences, Azna, IR Iran; 6 Imam Khomeini Hospital, Lorestan University of Medical Sciences, Borujerd, IR Iran; 7 School of Nursing, Lorestan University of Medical Sciences, Aligudarz, IR Iran

**Keywords:** Mental Health, Hypertension, C - Reactive Protein, Uric Acid

## Abstract

**Background:**

Multiple population-based human studies have established a strong association between increasing levels of serum C-reactive protein, uric acid and subsequent development of hypertension.

**Objectives:**

We aimed to investigate the association between mental well-being with presence of hypertension, hyperuricemia and hs-CRP levels. ‬‬

**Patients and Methods:**

This was a cross sectional study of 801 individuals aged 35-85 years old in Broujerd, Iran, included by randomized cluster sampling. General Health Questionnaire (GHQ-12) for assessing mental health/distress level, MONICA standard questions for evaluating hypertension history, serum hs-CRP and Serum Uric Acid (SUA) were evaluated Data were analyzed by appropriate statistical test such as chi-square, T-test and correlation.

**Results:**

One hundred eighty five patients (23.1%) had high distress/minor psychiatric disorders. SUA had significant association with hypertension (r = 0.64, P = 0.034). No significant relation was observed between hs-CRP and hypertension. The correlation between GHQ and hs-CRP was not significant but a weak and negative correlation was found between GHQ and SUA SUA (P = 0.012, r = -0.089).

**Conclusions:**

The weak and strong correlation among these parameters indicate that mental wellbeing relays on physical wellness and interact with each other; therefore, controlling hypertension along with uric acid control may effect mental health of any kind of patients.

## 1. Background

Psychological distress can be broadly defined as a negative interval state of the individual that is dependent on interpretation or appraisal of threat, harm or demand. Clinical epidemiologic studies have shown that psychological distress and related psychological factors such as depressive symptoms, anxiety and hostility are associated with cardiovascular disease progression (1). There is conflicting evidence regarding the association of hypertension with psychological distress, such as anxiety and depressive symptoms. The association may exist because of a direct effect of the raised blood pressure, adverse effects of treatment, or the consequences of labeling (Stigma). Several studies have reported positive associations; whereas others have reported weak or no associations (2-4). There is even some evidence to suggest lower blood pressure (BP) in participants with depressive or anxiety disorders. Blanchflower and Oswald (5) reported, cautiously, that blood pressure provides a biomarker for psychological well-being. Researches that examined the combined effects of hypertension and common mental disorders (such as anxiety and depression) on mortality in participants with both treated and untreated hypertension, demonstrated positive association between hypertension and total and CVD mortality when combined with common mental disorders (6). C-reactive protein (CRP) is a marker of low-grade inflammation, prospectively associated with increased risk of both cardiovascular disease and depression (7). Several prospective studies have demonstrated that High sensitive C - reactive protein (hs-CRP) is an independent predictor of future risk for cardiovascular events among healthy individuals, as well as patients with acute coronary syndromes. In addition, because half of all cardiovascular events occur in patients with low to moderate levels of low-density lipoprotein cholesterol, hs-CRP may aid in identifying patients at high risk for a first cardiovascular event who might otherwise be missed by lipid screening alone (8).‬‬ Hyperuricemia has also been found to be an independent risk factor for cardiovascular disease. Multiple population-based human studies have established a strong association between increasing levels of serum uric acid and subsequent development of hypertension (9). However, the relation between uric acid, inflammatory biomarkers and hypertension with mental wellbeing has not been assessed in a single context.

## 2. Objectives

In the present study, we aim assess mental well-being with presence of hypertension, hyperuricemia and hs-CRP.

## 3. Patients and Methods

### 3.1. Subjects

This was a cross sectional study on 801 individuals aged 35-85 years from Borujerd City, Lorestan, Iran in 2012. This is a descriptive cross sectional study, nested from a greater cohort regarding "healthy heart program". The included subject accounted 801 volunteers from all boroujerd's population. Greater Boroujerd city (Shahrestan-e-Boroujerd) was divided into 27 clusters (16 urban and 9 rural) according to municipality districts. Based on the actual population of 35 years-old and above in each cluster, 30-31 Broujerd's residents were included. The randomized sampling within each cluster was performed by randomization number according to building numbers. All participants signed an informed consent. Study tools were General Health questionnaire (GHQ-12) for evaluating mental health/distress level, MONICA standard questions for assessing hypertension history, and serum hs-CRP and Uric Acid (SUA). Other demographic and medical history information was obtained from their past medical record or the demographic checklist, prepared from this study. The socioeconomic state was evaluated based on the Iranian socioeconomic study regarding Tavassoli et al. study (10), using a researcher developed questionnaire consisting 7 components (place of residing, last educational degree, marital status, number of family members, net income, owning home and car and type of occupation). Its score rages between 6.5 and 14 (6.5-8.99 low, 9-11.49 medium and 11.5-14 high). Its Cronbach's alpha for our study was 0.894. All patients were informed about the study and signed consent was taken (Ethical code No. 1276, date of issue: Jan 11, 2011).

### 3.2. Factor Measurements

All patients underwent phlebotomy and 5 cc blood was contained into a vacutainer. The blood samples were stored in -4°C package and referred to our referral laboratory. Serum hs-CRP levels were assessed with immunoturbidimetric assay (Diagnostica kit, Germany). Inter-assay and intra-assay CVs were 3.2 and 0.9%. SUA was also measured by uric acid TOOS kit, Pars Azmoon Co, Iran. Total cholesterol, HDL and TG were measured by photometric assay with intra- and inter-assay CV less than 2% (Pars Azmoon Co, Iran). Blood pressure was measured by manual cuff Sphygmomanometer (Richter inc, Germany).The inter-observer Kappa coefficient was 0.774. General Health Questionnaire (GHQ) is a screening device for identifying minor psychiatric disorders in the general population. Suitable for all ages from adolescent upwards, it assesses the respondents, current state and asks if that differs from their usual state. It is therefore sensitive to short-term psychiatric disorders, but not too long-standing attributes of the respondent. GHQ-12 which is a quick, reliable and sensitive short form of questionnaire ideal for research studies was used in this study. In this questionnaire score o means healthy and GHQ ≥ 4 is considered as having high distress/minor psychiatric disorders (alpha chronbach = 0.87, Validity of 56% (11)). Furthermore, MONICA Manual which includes two questions related to the awareness and treatment of hypertension (health care worker informed and using medication regarding hypertension) were also asked from the subjects. For assessing coronary artery disease, Rose angina questionnaire and Minnesota ECG codes were used.

### 3.3. Statistical Analysis

Data were expressed as mean ± SD for quantitative variables and frequency (percentage) for categorical variables. Data was analyzed by SPSS (v. 20) using K-S test for assessing normality, chi-square for comparing percentages in two groups , t-test and Mann-Whitney-U test for comparing mean values between 2 groups with normal and non-normal distributed variables. Furthermore, Spearman correlation for assessing correlation between 2 quantitative variables, one way ANOVA and Bonferroni post hoc test for comparing mean values among 3 or more representative groups. The P value less than 0.05 were considered as significant.

## 4. Results

Eight hundred and one people participated in this study, 388 males and 413 females aged 35-85 years old. The mean age of participants was 54.8 ± 2.1 years. 592 participants were from urban areas and the rest were recruited from rural areas. Based on GHQ, 185 persons (23.1%) had high distress/minor psychiatric disorders (GHQ ≥ 4). 547 participants had high hs-CRP (> 3). Other demographic and analytic data are expressed in Table 1. Socioeconomic classification regarding GHQ score and other parameters are shown in Table 2.

**Table 1.** Variable in the 2 Groups Regarding GHQ-12 Results; Bold and Italic P Values are Significant (Mann-Whitney-U test)^a , b^

	Total	GHQ-12 < 3	GHQ-12 > 4	P Value
**Hypertension**	179 (22.3)	139 (22.4)	41 (22.2)	0.960
**Diabetes**	87 (10.9)	70 (11.4)	17 (9.2)	0.379
**Gender**				< 0.001
Male	388	321	61	
Female	413	288	125	
**Age, y**	54.82 ± 12.1	55.08 ± 12.0	53.94 ± 11.8	0.264
**Smoking**	253 (31.6)	185 (30)	68 (36.8)	0.111
**SE score**	10.51 ± 1.41	10.62 ± 1.39	10.17 ± 1.39	< 0.001
**Systolic BP, mmHg**	127.0 ± 21.5	127.5 ± 21.5	125.4 ± 21.3	0.253
**Diastolic BP, mmHg**	79.4 ± 11.7	80.0 ± 11.95	77.6 ± 11.0	0.018
**HS-CRP, IU**	6.14 ± 0.79	6.08 ± 0.74	6.30 ± 0.86	0.659
**SUA, mg/dL**	6.93 ± 3.71	7.01 ± 3.99	6.33 ± 2.44	0.028
**HDL, mg/dL**	44.11 ± 7.06	44.13 ± 6.70	44.00 ± 8.09	0.873
**LDL, mg/dL**	116.7 ± 38.8	116.2 ± 38.9	118.1 ± 38.3	0.563
**Total Cholesterol, mg/dL**	192.2 ± 40.7	192.3 ± 40.8	192.2 ± 40.4	0.982
**Triglyceride, mg/dL**	161.1 ± 112.1	162.6 ± 112.5	158.5 ± 110.7	0.665

^a^ Abbreviation: GHQ, general health questionnaire, SE, Socioeconomic; BP, blood pressure, SUA, serum uric acid; HDL, high density lipoprotein, LDL, low density lipoprotein.

^b^ Data are presented as Mean ± SD, No. (%).

**Table 2.** GHQ-12, Serum Uric Acid (SUA), High Sensitive CRP (hs-CRP) and Hypertension Frequency Regarding Socioeconomic Level of the Participants ^a,b,c^

Socioeconomic Levels	N	GHQ-12 score^ d^	SUA	hs-CRP^d^	Hypertension^e^
**Low**	79	2.91 ± 2.02a	6.34 ± 2.11	5.19 ± 5.5	25 (31)
**Medium**	486	2.47 ± 2.23b	6.86 ± 4.11	6.267.89	116 (23.8)
**High**	236	1.94 ± 2.00c	7.26 ± 2.98	6.218.75	31 (13.1)
**P Value**		0.001	0.534	0.136	0.001

^a^ Abbreviation: GHQ, general health questionnaire; SUA, serum Uric Acid; hs-CRP, high sensitive CRP.

^b^ Data are presented as Mean ± SD, No. (%).

^c^ Significant difference between "ac" and "bc", no other significant difference in post-hoc tests.

^d^ ANOVA test (bonferoni post-hoc).

^e^ Chi-square.

The SUA level was similar among rural and urban participants, but it was significantly different between two genders, higher in males. SUA had significant association with hypertension (r = 0.64, P = 0.034). It was higher in participants with hypertension. HS-CRP was compared among rural and urban participants, appeared that HS-CRP was similar among rural and urban population and also among males and females (P < 0.05). No significant relation was observed between hs-CRP and hypertension (P > 0.05). The association between high SUA and elevated distress levels (GHQ ≥ 4) was significant by t-test. To assess the relation between GHQ with hs-CRP and SUA the Spearman correlation test was used. The correlation between GHQ and hs-CRP was not significant (P = 0.602) but a weak and negative correlation was found between GHQ and SUA (P = 0.012, r = -0.089). In evaluating the relation between GHQ score and gender, we found that more women had GHQ ≥ 4 compared with men (30.1%, and 15.5% respectively, P < 0.001). Our results did not show any significant relation between gender and high hs-CRP (20.3% in females and 23.8% in males, P < 0.2850). The SUA was significantly lower in women than men (6.45 ± 2.5, and 7.33 ± 2.7 respectively, P < 0.001).

## 5. Discussion

The Present study was a cross sectional survey on 801 rural and urban population of Borujerd city. We evaluated the association between mental well-being, assessed by GHQ-12, presence of hypertension, SUA and hs-CRP in our study population; along with other demographic information. The strong association between hypertension and hyperuricemia has been recognized for more than a century. Mahomed, in his original description of essential hypertension (1879), noted that many of his subjects came from gouty families (12). Six large epidemiologic studies published over a few years have found that SUA predicts the later development of hypertension. One of these is the Normative Aging Study (13), which showed that the SUA independently predicts the development of hypertension when using age adjusted and multivariate models that include body mass, abdominal girth, alcohol abuse, serum lipid levels, plasma glucose level, and smoking status. Based on the prevalence of recent epidemiologic studies, it appears that an elevated SUA is an independent risk factor for kidney, disease, hypertension and cardiovascular diseases. Our findings are similar to these studies; as we found significant association between SUA and hypertension‬‬‬. Puustinen et al. conducted a prospective population-based study to evaluate the psychological distress could predict the development of the metabolic syndrome. No significant differences between the psychological distress groups were found regarding to educational status, smoking status, consumptions of alcohol, body mass index, hs-CRP levels, or intake of antihypertensive, cholesterol lowering, or anti-diabetic medication (14). This result is in concordance with our result that showed no significant relation between hypertension and hs-CRP. In a study on evaluating the association of hs-CRP with hypertension in some rural residents, Lee demonstrated that level of hs-CRP was not a risk factor for hypertension among adults aged over 50 years, living in a rural area (15). However Shafi Dar et al. reported a graded association between blood pressure and hs-CRP elevation in people with hypertension. Individuals with pre-hypertension or with shorter duration of hypertension (≤ 1 year, stage 0) had significantly a greater likelihood of hs-CRP elevation compared with chronic stage 1 or 2 hypertensive (16). Further studies should be performed to find the true nature of association between hs-CRP and hypertension. This study result showed that correlation between GHQ and hs-CRP was not significant (P = 0.602). Nazmi et al. (17) also found no association between CRP and minor psychiatric disorder in a representative sample of healthy young Brazilian adults. Almeida et al. also assessed the association between CRP concentration and depression in later life (18). The results of their study showed that the serum concentration of CRP is only weekly associated with depression scores in later life. CRP is a moderate predictor of coronary heart disease and, apart from periods of acute illness, its concentration remains relatively stable over time. Moreover, high CRP is associated with increased mortality in later life (19). The difference among various results may originate from difference baseline of mental status between countries or difference case setups. However, our negative findings are in contrast to some previous studies that have found significant associations between mental distress and elevated hs-CRP in young populations (20). More studies are required to shed light on these conflicting results. Previous studies have shown that uric acid (SUA) has strong anti-oxidant properties, and that high circulating levels of SUA are prospectively associated with improved cognitive performance in elderly people. We found a weak and negative correlation between GHQ-12 and SUA which means higher SUA is associated with better mental health. Almost similar result was found by Wu Y. His findings suggested that notwithstanding the associated increased risk of cardiovascular disease, SUA might play a protective role in aging-associated decline in muscle strength and cognitive function (20). Blanchflower et al. (5) in their study across different regions of UK evaluated the connection between the level of blood-pressure and the level of mental well-being. It builds on the intuition that, when under stress, human beings exhibit signs of strain on their cardiovascular system. They concluded, cautiously, that blood pressure provides a biomarker for psychological well-being. The difference among various results may originate from difference baseline of mental status between countries or difference case setups. Our results about the association between GHQ ≥ 4 and hypertension showed that the relation between hypertension and mental health in total study population (P = 0.62) and in patients with cardiac disease (P = 0.858) was not significant, but the relation was almost significant in healthy individuals (P = 0.058). Our result like above study implies that there is a possible relation between hypertension and mental health. Our results showed that gender, age, and blood pressure are associated with psychological distress and heart diseases. However, as the nature of a cross sectional study, we cannot assess the cause-effect direction within our variables. The weak and strong correlation among these parameters indicate that mental wellbeing relays on physical wellness and interact with each other; therefore, controlling hypertension along with uric acid control may effect mental health of any kind of patients. As our study was a population based research, the results can be generalized to cities similar to Broujerd in Iran and other developing countries; however, not having enough information regarding previous mental history and occult or undiagnosed inflammatory illnesses can hamper the quality of the results. Therefore, we suggest more specific studies on different populations to find more accurate evidence. Negative correlation between GHQ and SUA was found in this study, which means higher SUA is associated with better mental health. However, we found significant association between SUA and hypertension. Our findings also indicated that male gender and higher level of SUA had significant effects on stress level (GHQ ≥ 4). The relation between hypertension and mental health was almost significant in healthy individuals; therefore, for finding the real association between them and for considering hypertension as a marker for mental health, we recommend further prospective studies with larger sample size. We did not find significant relation between hypertension and hs-CRP.
